# Lennox-Gastaut syndrome: An overview

**DOI:** 10.4103/1817-1745.66666

**Published:** 2010

**Authors:** Ramnath Santhosh Ramanathan, Tina Ahluwalia, Ankush Sharma

**Affiliations:** Max Superspecialty Hospital, Saket, New Delhi, India

Sir,

A 7-year-old boy, a known case of GTCS (generalized tonic–clonic seizures) since 8 months of age whose seizures were poorly controlled with antiepileptic medications, was admitted with increased jerky movements of the body for the past few days. He had a history of birth asphyxia and delayed developmental milestones as well as cognitive impairment. On admission, the patient was conscious and hemodynamically stable. Cognitive impairment was present. He was moving all four limbs. He had an ataxic gait. He was able to hear and apparently had no vision problems. The plantars were bilaterally flexor.

**Figure 1:**
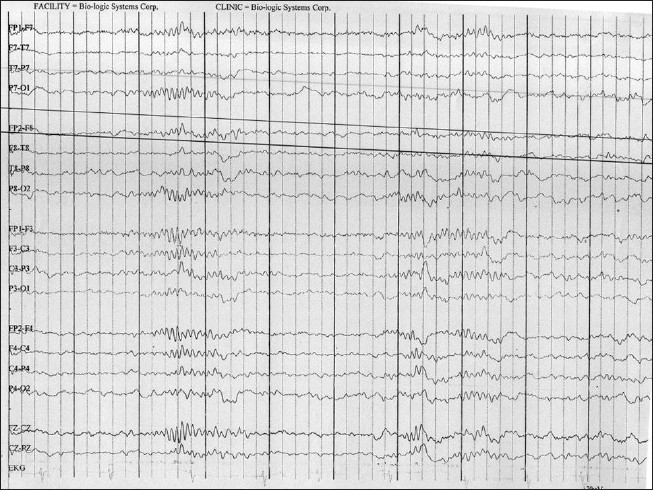
The 24 hour prolonged digital video EEG recorded from 02/02/2010 to 03/02/2010 recorded three events showing clinically head drop with EEG magnification of Pseudo-normalization lasting for 3-4 seconds. Interictal EEG record is suggestive of multifocal discharges

**Figure 2:**
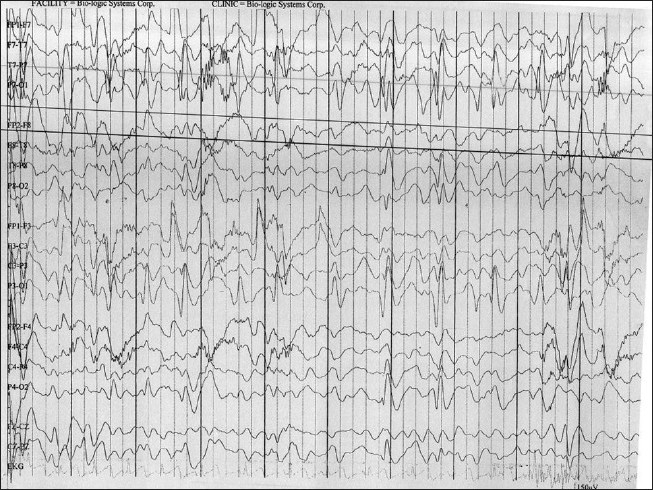
The 24 hour prolonged digital video EEG recorded from 02/02/2010 to 03/02/2010 recorded three events showing clinically head drop with EEG magnification of Pseudo-normalization lasting for 3-4 seconds. Interictal EEG record is suggestive of multifocal discharges

**Figure 3:**
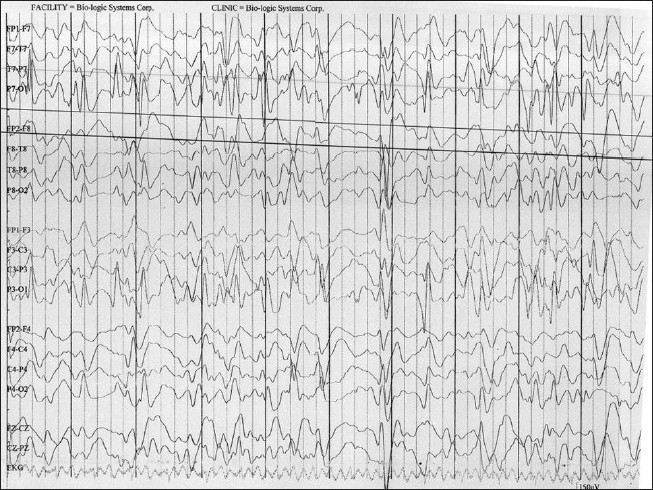
The 24 hour prolonged digital video EEG

**Figure 4:**
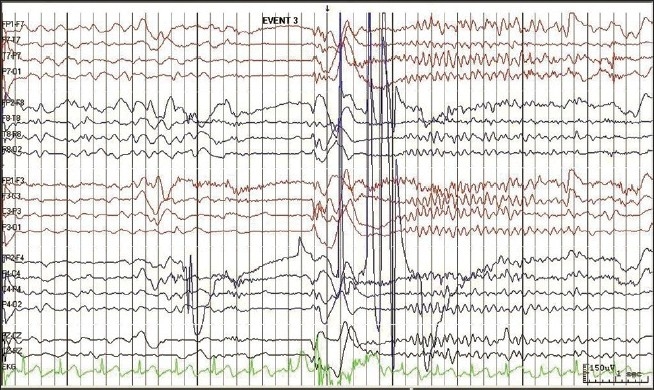
The 24 hour prolonged digital video EEG recorded from 02/02/2010 to 03/02/2010 recorded three events showing clinically head drop with EEG magnification of Pseudo-normalization lasting for 3-4 seconds. Interictal EEG record is suggestive of multifocal discharges

**Figure 5:**
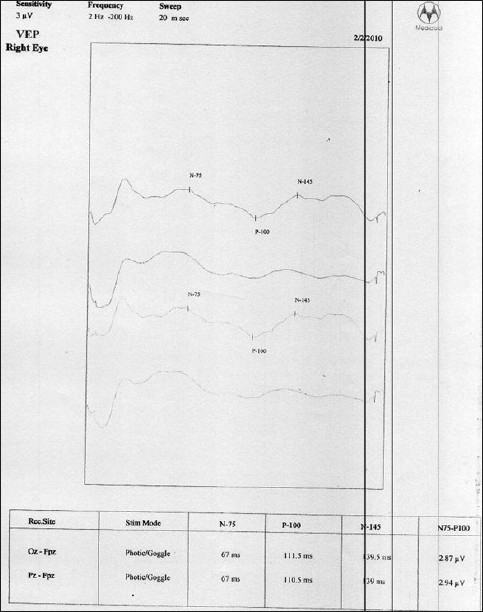
VEP showed bilateral prolonged P100 latency

**Figure 6:**
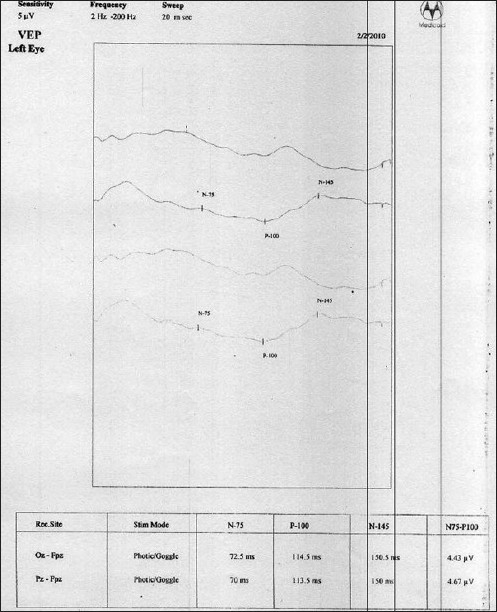
VEP showed bilateral prolonged P100 latency

MRI showed no focal lesion and a normal hippocampus bilaterally. A 24-hour prolonged digital video EEG [Figures 1–4] recorded three events of clinical head drop accompanied by electroencephalgraphic (EEG) magnification of pseudonormalization, each lasting for 3–4 seconds. The interictal EEG record was suggestive of multifocal spike-and-wave discharges in both hemispheres, particularly in the left frontocentral, right parieto-temporo-occipital region, and left parieto-occipital region, with intermittent independent generalized polyspike wave discharge and intermittent <3-Hz generalized spike-and-wave discharges lasting 1–8 seconds. The VEP (visual evoked potential) [Figure 5, 6] report showed bilateral prolonged P100 latency and decreased amplitude. The BAER (brainstem audiometry-evoked response) study was normal bilaterally. The tandem mass spectroscopy report was negative. All routine blood investigations were normal. The patient was managed with syrup valproate, with tablet lamotrigine added later. Satisfactory control of seizures was achieved.

The Lennox-Gastaut syndrome (LGS) is classified as an epileptic syndrome characterized by the presence of various types of generalized seizures (tonic, atonic, and atypical absences) that appear at a certain age (1–8 years), with an interictal EEG showing an abnormally slow basic rhythm interrupted by slow spike-and-wave complexes (<3 Hz) and progressive mental deterioration.[1] It accounts for only 2%–5% of childhood epilepsies. LGS is a severe form of childhood epilepsy, characterized by multiple seizures and cognitive impairment.

To date, there are no known laboratory investigations to aid in the diagnosis of LGS. Neuroimaging has an important role to play in the search for the underlying etiology of LGS. Abnormalities associated with LGS that have been revealed by neuroimaging include tuberous sclerosis, brain malformations (e.g., cortical dysplasias), hypoxia-ischemia injury and frontal lobe lesions. The diagnosis depends on the presence of the specific EEG findings. Prolonged video EEG should be performed to record both awake and sleep EEG to assist in confirming a suspected diagnosis and to capture and classify each of the patient’s multiple seizure types. The hallmark of the awake interictal EEG in patients with LGS is the diffuse slow spike wave. This pattern consists of bursts of irregular and generalized spikes or sharp waves followed by a sinusoidal 35- to 400-millisecond slow wave with an amplitude of 200–800 *μ*V, which can be symmetric or asymmetric. The frequency of the slow spike wave activity is commonly 1.5–2.5 Hz. The optimum treatment for LGS remains uncertain and no study to date has shown any one drug to be highly efficacious. Rufinamide, lamotrigine, topiramate and felbamate may be helpful as add-on therapy.[2] Valproate, used alone, produces a decrease in seizures (of greater than 50%) in 25%–30% of patients. In late 2008, the Food and Drug Administration approved rufinamide for adjunctive use in the treatment of seizures associated with LGS, which makes it the first new antiepileptic drug to be approved for use in the pediatric age-group prior to approval for use in adults.[3] Co-administration of valproate decreases rufinamide clearance, thus requiring dose adjustment. The simulations support the proposal for a lower maximum daily rufinamide dose for patients under 30 kg receiving both drugs: a dose of 600 mg/day is proposed as a maximum daily dose in children who are also receiving valproate concomitantly whereas, in the absence of valproate, the maximum daily dose is 1000 mg/day.[4] Lastly, resective epilepsy surgery should be considered for children with LGS, even though there may be abundant generalized and multiregional EEG abnormalities.[5]
